# Feasibility of using plasma p‐tau217 as a surrogate for brain Aβ PET in the community

**DOI:** 10.1002/alz.092551

**Published:** 2025-01-09

**Authors:** Wasiu Gbolahan Balogun, Rebecca A Deek, Xuemei Zeng, Anuradha Sehrawat, Yijun Chen, Pamela C.L. Ferreira, Bruna Bellaver, Tara K Lafferty, Guilherme Povala, M. Ilyas Kamboh, Victor L Villemagne, William E Klunk, Tharick A. Pascoal, Mary Ganguli, Oscar L. Lopez, Ann D Cohen, Beth E. Snitz, Thomas K Karikari

**Affiliations:** ^1^ University of Pittsburgh, Pittsburgh, PA USA; ^2^ University of Pittsburgh Alzheimer's Disease Research Center, Pittsburgh, PA USA; ^3^ University of Pittsburgh School of Medicine, Pittsburgh, PA USA

## Abstract

**Background:**

Plasma p‐tau217 is probably the best‐performing blood biomarker to detect brain amyloid‐beta (Aβ) accumulation. However, previous studies were mostly performed in highly selected cohorts. Thus, it is unclear if it can be used independently of confirmatory neuroimaging tests to identify individuals with abnormal brain Ab load in the wider population where positron emission tomography (PET) may be unavailable. We evaluated the feasibility of plasma p‐tau217 replacing Ab PET in two population‐based cohorts.

**Methods:**

This study used data from two population‐based cohorts; the Monongahela‐Youghiogheny Health Aging Team (MYHAT) Neuroimaging study (n=111, median age 76 IQR: 72‐80, 54% female), in a medically and economically‐depressed Rust Belt area of southwestern Pennsylvania, and the Human Connectome Project (HCP) (n=229, median age was 62 IQR: 56‐70, 65% female) of self‐identified Black and non‐Hispanic Whites in Pittsburgh city. Plasma p‐tau217 was measured using assays from the University of Pittsburgh, ALZpath Inc., and NULISA from Alamar Biosciences. Aβ imaging was performed with ^11^C‐PiB, where global Aβ+ was defined using a threshold of 1.346 SUVR.

**Results:**

The primary analysis used ALZpath p‐tau217 for both studies. Specifically, MYHAT‐NI reported a Positive Predictive Value (PPV) of 0.6 and Negative Predictive Value (NPV) of 0.92, as well as sensitivity and specificity of 0.82 and 0.80, respectively. PPV measures the probability a predicted positive patient has Ab accumulation, NPV is defined analogously for negative patients. Secondary subset analyses using the UPitt p‐tau217 and NULISA p‐tau217 assays showed similar performances (PPV: 0.56‐0.60, NPV: 0.89‐0.93). The primary analysis in HCP showed a PPV of 0.40 and NPV of 0.97, with corresponding sensitivity of 0.85 and specificity of 0.78. The secondary analysis using UPitt p‐tau217 performed comparably (PPV: 0.38 and NPV: 0.97).

**Conclusion:**

The consistently high NPV but poor PPV suggest that all the plasma p‐tau217 assays will be most useful as a first‐screening tool to identify individuals without pathological evidence of brain Ab for exclusion from clinical trials, therapeutic trials, and prescription of approved therapeutic agents. This would enable streamlined enrichment of individuals who would require confirmatory testing. Future research will investigate impacts of population health outcomes including comorbidities.

